# A Conceptual Framework for Drift-Aware IMU-Based Gait Analysis Using Motion Capture References

**DOI:** 10.3390/s26206657

**Published:** 2026-10-21

**Authors:** Eugene Rezk

**Affiliations:** 1 Virtual and Augmented Reality Group, Institute for Visual Computing and Human-Centered Technology, Faculty of Informatics, TU Wien, 1060 Vienna, Austria; eugene.rezk@medunigraz.at; 2 U089 Entrepreneurship in Digital Health (EDITH), Medical University of Graz, 8010 Graz, Austria; 3 Department of Physics and Biophysics, Medical University of Graz, 1210 Graz, Austria; 4 Department of Orthopedics and Traumatology, Military Hospital Vienna, 1120 Vienna, Austria; 5 Department of Trauma Surgery, Trauma Center Vienna, Meidling Site, 1020 Vienna, Austria; 6 Faculty of Medicine, Sigmund Freud Private University Vienna, 1020 Vienna, Austria

**Keywords:** inertial measurement unit (IMU), gait analysis, drift correction, zero-velocity update (ZUPT), extended Kalman filter (EKF), motion capture, residual analysis, telemedicine, wearable sensors

## Abstract

Inertial measurement unit (IMU)-based gait analysis represents a promising modality for telemedical assessment; however, integration drift remains a fundamental limitation for reliable long-term motion estimation. In this perspective, we propose a conceptual framework for drift-aware IMU-based gait reconstruction that combines established drift-mitigation techniques with phase-dependent analysis of reconstruction residuals. The framework envisages comparing IMU-derived trajectories with motion-capture-derived reference trajectories to quantify reconstruction errors and characterize their temporal and gait-phase-dependent structure in future validation studies. It is hypothesized that these residuals may exhibit structured, gait-phase-dependent patterns arising from multiple sources, including sensor-related errors and biomechanical variability. Drift is therefore treated primarily as a technical error source, while any association between residual error patterns and gait stability or motor-control characteristics remains a hypothesis requiring future validation. The framework outlines a potential approach for integrating wearable inertial sensing, motion-capture-based reference data, and probabilistic filtering techniques. It may provide a conceptual foundation for future telemedical gait assessment systems incorporating phase-dependent analysis of reconstruction errors.

## 1. Introduction

**Figure 1:**
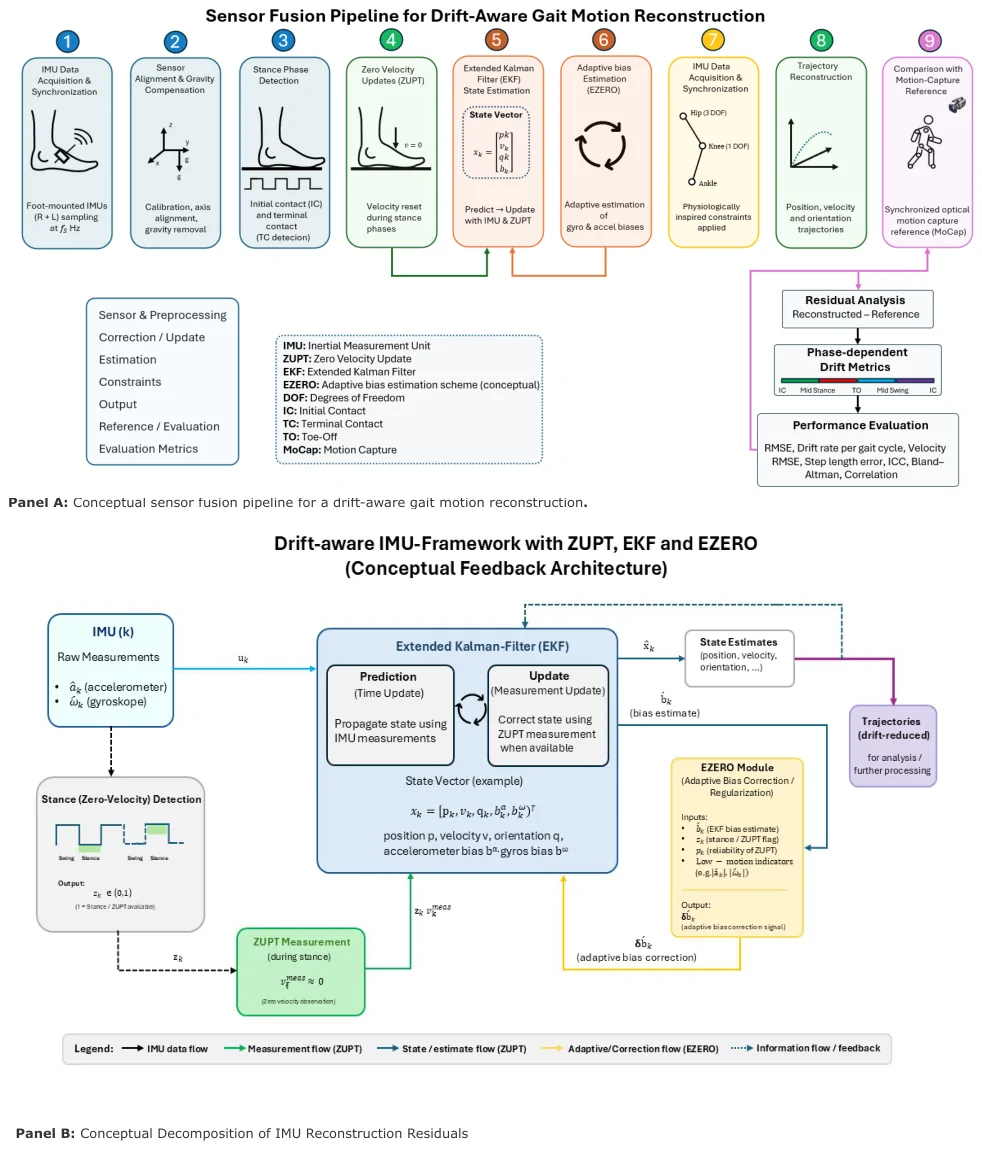
(**Panel A**): Conceptual architecture of the proposed drift-aware IMU gait reconstruction framework. The framework comprises sequential stages for synchronized IMU acquisition, preprocessing, stance-phase detection, zero-velocity updates (ZUPT), extended Kalman filtering (EKF), adaptive bias estimation (EZERO), biomechanical constraint enforcement, and trajectory reconstruction. Motion-capture-derived reference trajectories are proposed for subsequent quantitative evaluation of reconstruction error and its gait-phase-dependent structure. The framework is conceptual and does not represent an experimentally validated implementation. (**Panel B**): The diagram illustrates the proposed interaction between ZUPT-based velocity observations, EKF state estimation, and the adaptive EZERO bias-correction mechanism. ZUPT provides zero-velocity information during detected stance phases, which is incorporated into the EKF to update the estimated kinematic state and inertial bias. EZERO is conceptualized as an auxiliary adaptive mechanism that uses the EKF bias estimate to constrain and stabilize its temporal evolution, thereby providing an adaptive bias-correction signal for subsequent EKF state estimation. The diagram represents the proposed methodological architecture and does not depict an experimentally validated implementation.

In recent years, inertial measurement units (IMUs) have become an important technology for telemedicine and wearable health monitoring due to their portability, low cost, and ability to capture motion in unconstrained environments [1,2,3,4,5]. In particular, IMU-based gait analysis has emerged as a viable alternative to laboratory-based motion capture systems, enabling continuous assessment of locomotion in real-world settings [6,7,8]. Gait analysis plays a crucial role in the evaluation of neurological, musculoskeletal, and age-related disorders, where deviations in spatiotemporal parameters and movement variability may serve as clinically relevant biomarkers [9,10,11]. Despite these advantages, accurate estimation of kinematic parameters from IMU data remains challenging due to sensor noise, measurement bias, orientation inaccuracies, and numerical integration errors, which can lead to drift that accumulates over time and degrades position and velocity estimates [4,5]. Conventional approaches combine inertial measurements with filtering techniques, including Kalman and complementary filters, to improve motion estimation [12,13]. In foot-mounted IMU systems, zero-velocity updates (ZUPT) are widely adopted to constrain velocity estimates during detected stance phases, when foot velocity is assumed to be approximately zero [8]. The validity of wearable IMU-based gait analysis depends on the biomechanical parameter being estimated, with systematic reviews examining lower-limb range of motion and estimated ground reaction forces against reference measurements [14]. Although these methods can reduce accumulated errors, their performance depends on reliable gait-event detection, sensor calibration, appropriate filter design, and the validity of the underlying motion assumptions [6]. Data-driven approaches and advanced sensor fusion strategies have also been investigated to improve robustness, although their reliance on annotated datasets and potential limitations in interpretability may pose challenges in some clinical applications [9,15]. An additional challenge is that the error in a reconstructed gait trajectory is not necessarily equivalent to inertial drift. Differences between IMU-derived estimates and external reference trajectories may arise from several interacting sources, including sensor bias, orientation and alignment errors, synchronization inaccuracies, gait-event detection, filter behavior, and deviations from assumed biomechanical constraints. Consequently, reconstruction residuals require careful interpretation and should not be attributed to inertial drift alone. In particular, the extent to which residual errors exhibit gait-phase-dependent structure and whether such patterns reflect technical estimation errors, movement-related characteristics, or a combination of both remain to be investigated [4,5]. Optical motion capture systems provide an external reference for evaluating IMU-derived kinematic estimates under controlled measurement conditions [7,16,17]. Synchronized inertial and motion-capture measurements can enable the quantitative comparison of reconstructed trajectories and the characterization of residual errors across gait phases. However, such residuals may reflect both measurement-system discrepancies and limitations of the reconstruction model; therefore, their interpretation requires consideration of sensor calibration, coordinate-frame alignment, temporal synchronization, and biomechanical assumptions. This motivates a framework that considers inertial state estimation, adaptive bias correction, biomechanical constraints, and reference-based residual analysis within a common methodological architecture. Rather than introducing replacements for established methods such as ZUPT or the extended Kalman filter (EKF), the proposed framework explores their integration with an auxiliary adaptive bias-estimation mechanism, referred to as EZERO. EZERO is conceptualized as a mechanism that uses EKF-derived bias estimates to constrain and stabilize their temporal evolution and to provide an adaptive bias-correction signal. Its mathematical formulation, parameterization, and interaction with the EKF require explicit specification and empirical evaluation. The framework also proposes the analysis of gait-phase-dependent reconstruction residuals to investigate their structure, while treating the potential presence of movement-related information as a hypothesis rather than an established finding. The objective of this Perspective is to present a conceptual architecture for drift-aware IMU-based gait reconstruction and reference-based residual analysis, describe the proposed interaction between established inertial estimation methods and the EZERO mechanism, and identify methodological considerations for implementation and validation. The framework is intended primarily to support the investigation of gait in healthy individuals and populations with sufficiently preserved locomotor function. Its applicability to severe pathological gait, where abnormal or highly variable movement patterns may violate common biomechanical assumptions, remains an open question requiring dedicated evaluation. Figure 1 summarizes the proposed architecture and the conceptual interaction between its main components.

## 2. Design

The proposed framework is structured around three methodological components: (i) biomechanically informed gait modeling and reference trajectory generation; (ii) sensor emulation and, in future validation studies, integration of experimentally recorded inertial data; and (iii) drift-aware motion reconstruction [12,13]. The overall design is intended to provide a structured basis for investigating drift behavior and reconstruction strategies under both controlled and experimentally recorded conditions [4,15]. In the present perspective, these components define the proposed methodological architecture rather than constituting a completed quantitative validation study.

### 2.1. Gait Modeling and Reference Trajectories

A biomechanically inspired periodic gait model is proposed to represent foot motion during level walking [12,13]. Within the proposed framework, this model serves as a simplified and controlled representation for illustrating drift mechanisms and defining reproducible conditions for methodological evaluation. The conceptual trajectory captures the characteristic alternation between stance and swing phases of human gait [8]. Horizontal motion represents forward progression, whereas vertical motion represents foot clearance during the swing phase. These components can be represented as smooth periodic signals parameterized by predefined step length, cadence, and foot clearance. Such a simplified representation facilitates controlled methodological analysis but does not reproduce the variability and biomechanical complexity of real human locomotion [5]. For empirical evaluation, the proposed framework may be applied to experimentally recorded datasets containing inertial measurements and motion-capture-derived reference trajectories. Publicly available datasets, such as OxIOD, may provide suitable resources, depending on their specific sensor configurations, motion-capture protocols, and availability of the reference variables required for the proposed analysis [7,16]. In future validation studies, simulated and experimentally recorded data could be used complementarily to investigate gait-phase-dependent residual characteristics and assess the behavior of different drift-compensation strategies under controlled and realistic conditions [6]. This combination is intended to support a progression from controlled methodological investigation toward empirical evaluation while maintaining potential relevance for telemedical applications [9].

### 2.2. Sensor Emulation and Data Integration

Within the proposed framework, synthetic IMU signals can be generated from reference trajectories by incorporating representative sensor characteristics, including measurement noise, bias, and drift effects [4,5,18]. In particular, time-varying bias components can be modeled to represent stochastic behavior typically observed in MEMS-based inertial sensors [18,19]. Such synthetic signals are intended to provide controlled and reproducible conditions for investigating the mechanisms contributing to inertial drift. Measured acceleration can be represented as the superposition of the underlying acceleration signal, a time-varying bias component, and stochastic measurement noise, as expressed in Equation (1).

(1) $$a_{\mathrm{meas}}\left(t\right)=a_{\mathrm{true}}\left(t\right)+b\left(t\right)+n\left(t\right)$$

In addition to synthetic data, the proposed framework may subsequently be evaluated using experimentally recorded datasets to capture natural motion variability and sensor-specific characteristics. Publicly available datasets such as OxIOD may provide suitable resources for this purpose, depending on the available sensor configuration and reference measurements [7,16]. Datasets containing synchronized inertial measurements and motion-capture-derived reference trajectories could enable comparisons between IMU-derived estimates and an external reference. The combination of synthetic and experimentally recorded data is therefore proposed as a methodological strategy for progressing from controlled analysis toward empirical validation. In future studies, this approach could be used to investigate phase-dependent residual characteristics, compare drift-compensation strategies, and assess the extent to which observed residual structures persist under increasingly realistic motion conditions [6].

### 2.3. Reference Dataset and Experimental Configuration

For future empirical validation, the multi-sensor human gait dataset published by Santos et al. is proposed as a potential reference dataset. It comprises synchronized inertial measurements and optical motion-capture data from 25 adults without lower-limb injuries, recorded during level walking at a self-selected pace [20]. The dataset includes accelerometer and gyroscope measurements from a custom IMU attached to the leg, together with three-dimensional trajectories obtained using optical motion capture. The synchronized recordings provide a basis for comparing IMU-derived motion estimates with external reference trajectories. Before analysis, the available data and their suitability for the intended reconstruction tasks will need to be verified. Future implementations should explicitly document coordinate-system alignment, gravity compensation, sensor placement, temporal synchronization, and gait-phase or event-label generation. These procedures are essential for distinguishing inertial drift from other sources of reconstruction error, including calibration, orientation, synchronization, and modelling errors. The present perspective includes a limited descriptive inspection of raw gyroscope measurements from a representative recording in this dataset, as detailed in Section 3. However, it does not report quantitative trajectory reconstruction, formal sensor-bias characterization, or empirical validation of the proposed framework. The dataset is therefore considered a potential basis for future systematic evaluation of the framework.

### 2.4. Sensor Placement and Calibration Considerations

The accuracy of IMU-based motion reconstruction is influenced by sensor orientation and placement. Misalignment between the sensor coordinate system and the anatomical reference frame may introduce systematic estimation errors that propagate through subsequent orientation estimation and trajectory reconstruction. The selected dataset includes inertial sensors attached to the participant’s leg, providing a defined experimental configuration for the proposed validation. However, the sensor placement and coordinate-system alignment used in the original recordings must be considered when interpreting the resulting estimates. For potential home-based telemedical applications, a functional calibration procedure may be required to reduce sensitivity to user-dependent sensor attachment. Such a procedure could include a short static standing phase followed by predefined calibration movements, enabling estimation of the sensor orientation relative to the corresponding body segment. Future implementations may additionally incorporate automatic calibration routines to improve robustness against variability in sensor attachment and placement. The effectiveness of these calibration strategies would require separate empirical evaluation under controlled and real-world placement conditions.

### 2.5. Sensor Fusion Pipeline

The proposed motion reconstruction framework combines Zero Velocity Updates (ZUPT), an extended Kalman filter (EKF), and a conceptual adaptive bias-estimation component (EZERO). These components are intended to serve complementary functions: ZUPT provides velocity observations during detected stance phases, the EKF propagates and updates the estimated kinematic state, and EZERO is proposed as a reliability-aware mechanism for informing bias estimation from suitable low-motion intervals. The resulting state estimates could be compared with motion-capture-derived reference trajectories in future validation studies to characterize reconstruction residuals and investigate drift-compensation behavior.

#### 2.5.1. Extended Kalman Filter Formulation

The EKF state vector is defined as

$$x_{k}={\left[\begin{array}{cccc} p_{k} & v_{k} & q_{k} & b_{k} \end{array}\right]}^{T},$$

where $p_{k}$ denotes position, $v_{k}$ is velocity, $q_{k}$ orientation is represented by a quaternion, and $b_{k}$ is the estimated inertial sensor bias state.

The process model follows a general nonlinear inertial kinematic formulation, i.e.,

$$x_{k+1}=f\left(x_{k},a_{k},\omega_{k}\right)+w_{k},$$

where $a_{k}$ and $\omega_{k}$ denote the measured specific force and angular velocity, respectively, and $w_{k}$ represents process noise. The nonlinear state-transition function (f(·)) describes the propagation of position, velocity, orientation, and bias states between consecutive time steps.

During detected stance phases, zero-velocity observations can be incorporated into the filter using the measurement model:

$$z_{k}=H_{\mathrm{ZUPT}}x_{k}+\nu_{k},$$

where $z_{k}=0$ represents the zero-velocity observation, $H_{\mathrm{ZUPT}}$ denotes the observation matrix that selects the velocity components from the state vector such that $H_{\mathrm{ZUPT}}x_{k}=v_{k}$, and $\nu_{k}$ represents measurement noise. For a state vector containing position, velocity, orientation, and inertial bias, the observation matrix can be expressed schematically as

$$H_{\mathrm{ZUPT}}=\left[\begin{array}{cccc} 0_{3\times 3} & I_{3\times 3} & 0_{3\times 4} & 0_{3\times n_{b}} \end{array}\right],$$

where $I_{3\times 3}$ is the identity matrix and $n_{b}$ denotes the dimension of the bias state. Thus, the observation matrix selects the velocity components rather than multiplying the state vector by zero. Substituting $z_{k}=0$ and $H_{\mathrm{ZUPT}}x_{k}=v_{k}$ into the measurement model gives

$$0=v_{k}+\nu_{k}.$$

This equation represents a zero-velocity measurement subject to measurement uncertainty; it does not imply that the estimated velocity must already be zero before the filter update. The innovation, defined as the difference between the observation and the predicted observation, is

$$\iota_{k}^{\mathrm{ZUPT}}=z_{k}-H_{\mathrm{ZUPT}}{\hat{x}}_{k|k-1}=-{\hat{v}}_{k|k-1},$$

where ${\hat{x}}_{k|k-1}$ and ${\hat{v}}_{k|k-1}$ denote the predicted state and velocity estimates before the ZUPT measurement update, respectively. The EKF uses this innovation, together with the estimated state uncertainty and measurement noise covariance, to correct the state estimate.

The corresponding zero-velocity constraint is expressed as

(2) $$v_{k}\approx 0,\;k\in T_{\mathrm{stance}},$$

where $T_{\mathrm{stance}}$ denotes the set of time indices classified as stance phases [8,18].

The process and measurement noise covariance matrices (Q and R) characterize uncertainty in state propagation and ZUPT observations, respectively. Their specification depends on sensor characteristics, motion conditions, and the reliability of stance-phase detection. Consequently, these covariance matrices should be defined according to the selected sensor configuration and evaluated through sensitivity analyses in future implementations. In particular, Q should reflect uncertainty in inertial state propagation, whereas R should account for uncertainty in the zero-velocity observations. The effects of these choices on reconstruction stability and residual characteristics remain to be assessed empirically.

##### Reliability-Aware Bias Estimation and Residual Pattern Analysis

Beyond state estimation and drift mitigation, the proposed framework considers whether reconstruction residuals may contain information relevant to the characterization of gait abnormalities. It distinguishes between reliability-aware bias estimation, which aims to account for the suitability of candidate measurement intervals, and residual pattern analysis, which examines reconstruction errors in relation to gait phases and independently assessed clinical or biomechanical characteristics. These functions are analytically distinct: Technical errors and genuine movement characteristics may produce overlapping residual patterns, while overly aggressive drift correction could suppress movement-related information of potential clinical interest. Accordingly, the framework does not assume that reconstruction residuals are inherently pathology-specific. Instead, it provides a conceptual basis for investigating phase-dependent residual patterns after relevant technical error sources have been characterized. The mechanisms for bias adaptation, residual feature extraction, and assessment of potential clinical associations remain to be specified and evaluated in future studies.

##### Adaptive Bias Estimation (EZERO)

The proposed EZERO component is conceived as an auxiliary mechanism for reliability-aware adaptation of inertial bias estimates within the sensor-fusion framework. Whereas the extended Kalman filter (EKF) propagates and updates the estimated kinematic state and its associated bias states, EZERO is intended to assess whether information from candidate low-motion intervals is sufficiently reliable to inform bias estimation. This assessment could consider inertial measurements and available motion-state or gait-phase information, as conceptually expressed by

(3) $$r_{k}=g_{\mathrm{EZERO}}\left(\omega_{1:k}^{m},a_{1:k}^{m},s_{1:k}\right),\;r_{k}\in \left[0,1\right],$$

where $r_{k}$ denotes a conceptual reliability score; $g_{\mathrm{EZERO}}\left(\cdot \right)$ represents an assessment function that remains to be specified; $\omega_{1:k}^{m}$ and $a_{1:k}^{m}$ denote the gyroscope and accelerometer measurements, respectively, up to time step *k*; and $s_{1:k}$ represents available information on detected motion states or gait phases. The score is intended to reflect the suitability of a candidate interval for bias estimation; it does not establish that the sensor is stationary.

The resulting reliability assessment could inform a subsequent bias-estimation update, represented conceptually as

(4) $${\hat{b}}_{k}=U\left({\hat{b}}_{k-1},y_{k}^{\mathrm{bias}},r_{k}\right),$$

where ${\hat{b}}_{k-1}$ and ${\hat{b}}_{k}$ denote the bias estimates before and after the update, respectively; $y_{k}^{\mathrm{bias}}$ represents information available for bias estimation; and U(·) denotes an update function that remains to be formally defined.

**Figure 2:**
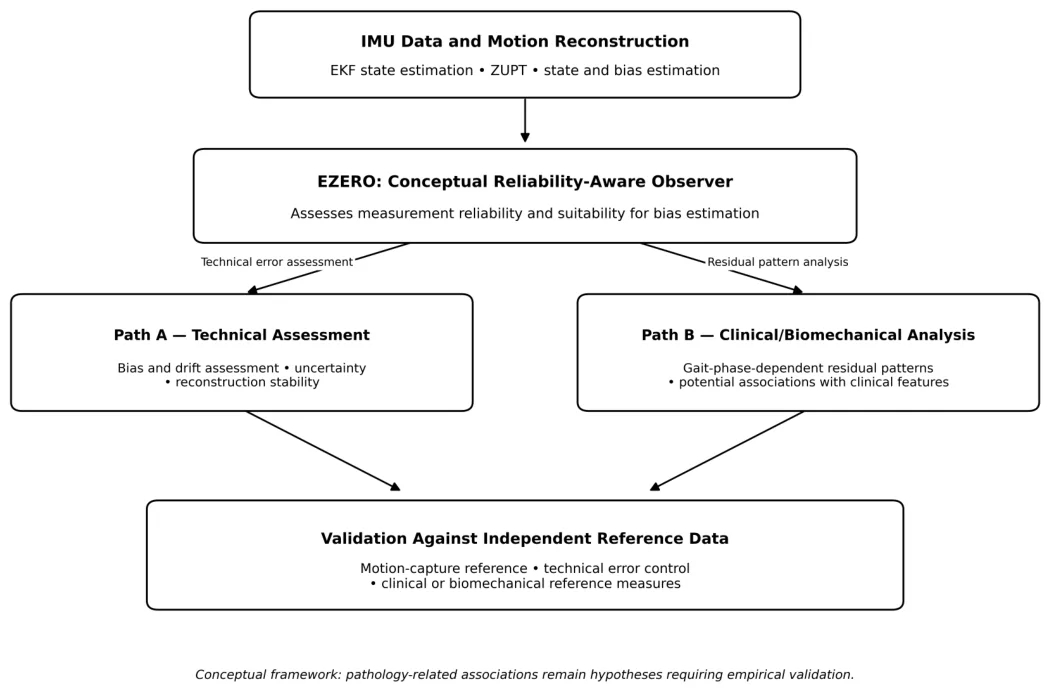
Conceptual overview of the proposed EZERO framework for reliability-aware inertial bias estimation and its relationship to EKF-based state estimation and gait-phase-dependent residual analysis. The framework illustrates the intended conceptual relationships between inertial measurements, reliability assessment of candidate low-motion intervals, bias estimation, and trajectory reconstruction. Comparison with motion-capture reference trajectories enables the subsequent analysis of reconstruction residuals across gait phases. The diagram represents a proposed framework; the EZERO update mechanism and its effectiveness have not yet been formally implemented or empirically validated.

A key consideration is that a detected gait stance phase does not necessarily imply zero angular velocity of the sensor. Stance detection alone therefore does not establish that a zero-angular-rate constraint is valid. A future implementation would need to define explicit criteria for identifying suitable intervals and specify how uncertainty in their classification affects the bias update or associated measurement covariance. The interaction between EZERO and the EKF would also need to be formulated to avoid redundant bias estimation or repeated use of the same measurement information. Thus, EZERO represents a conceptual reliability-aware component rather than a fully specified adaptive bias-estimation algorithm. Its update rule, adaptation parameters, uncertainty model, and interaction with the EKF remain to be defined. No claim is made that EZERO has been implemented, reduces inertial drift, or outperforms established bias-estimation approaches. Formal algorithm development, comparison with appropriate baseline methods, and empirical evaluation are required to determine its feasibility and potential contribution to drift-aware gait reconstruction. The intended contribution of EZERO is not the introduction of a new state estimator in itself, but the conceptual integration of reliability-aware selection of candidate low-motion intervals with subsequent gait-phase-dependent reconstruction residual analysis. This distinguishes the proposed framework from approaches concerned primarily with state estimation or inertial bias compensation. Whether this integration provides benefits beyond established bias-estimation methods remains to be determined through comparative evaluation. The conceptual relationships between reliability assessment, bias estimation, EKF-based state estimation, and gait-phase-dependent residual analysis are illustrated in Figure 2.

##### Trajectory Reconstruction and Residual Analysis

Following motion reconstruction, the estimated IMU-based trajectory can be compared with a synchronized motion-capture reference trajectory to characterize reconstruction accuracy [7,16]. The position residual at time step *k* is defined as

(5) $$e_{k}={\hat{p}}_{k}^{\mathrm{IMU}}-p_{k}^{\mathrm{MoCap}},$$

where ${\hat{p}}_{k}^{\mathrm{IMU}}$ denotes the reconstructed position obtained from the IMU-based processing pipeline and $p_{k}^{\mathrm{MoCap}}$ denotes the corresponding reference position derived from motion capture. This comparison presupposes appropriate temporal synchronization and coordinate-system alignment.

The resulting residual represents the combined discrepancy between the reconstructed and reference trajectories. It may arise from multiple sources, including inertial sensor bias, integration drift, calibration and orientation errors, temporal synchronization, coordinate-frame misalignment, gait-event detection, and biomechanical modelling assumptions [4]. Consequently, the residual should not be interpreted as a direct measure of inertial drift alone. It may also be influenced by the characteristics of the movement being reconstructed; technical reconstruction errors and genuine movement characteristics therefore need to be distinguished when interpreting residual patterns.

##### Gait-Phase-Dependent Residual Features and Potential Clinical Associations

Beyond global reconstruction-error measures, the temporal structure of the residuals may be examined across gait phases [5,6]. Let the residual features for participant *i* and gait phase *j* be represented by

(6) $$\phi_{i,j}=\Psi \left(\left\{e_{k}:k\in G_{i,j}\right\}\right),$$

where $G_{i,j}$ denotes the set of time indices assigned to gait phase *j* for participant *i* and Ψ(·) represents a feature-extraction function that remains to be defined. Potential features could characterize residual magnitude, variability, temporal structure, or axis-specific patterns. Their selection, reliability, and sensitivity to technical error sources would need to be specified and evaluated in future studies. The resulting residual features may then be investigated for associations with independently assessed clinical or biomechanical characteristics, denoted by $c_{i}$. This research question can be represented schematically as

(7) $$\phi_{i,j}\;⟷\;c_{i},$$

where the bidirectional arrow denotes a potential association to be investigated rather than an established relationship or causal effect. Such analyses would require appropriate clinical or biomechanical reference measures and consideration of relevant technical error sources. In particular, any potential pathology-related interpretation would need to be distinguished from effects attributable to sensor bias, integration drift, synchronization, coordinate-system alignment, or other reconstruction errors. The framework therefore proposes to investigate whether reproducible, gait-phase-dependent residual patterns persist after relevant technical error sources have been characterized or controlled and whether these patterns are associated with independently assessed movement characteristics. It does not assume that residual patterns are inherently pathology-specific or that they provide diagnostic information. The residual feature-extraction procedure and the statistical assessment of potential clinical associations remain to be formally specified and empirically validated.

### 2.6. Baseline and Hybrid Reconstruction Approaches

The proposed framework considers two complementary reconstruction approaches: (i) a naive integration baseline and (ii) a hybrid drift-aware reconstruction framework [8,12,13]. These approaches provide a conceptual basis for future comparative evaluation of inertial reconstruction and drift-compensation strategies. As the framework has not yet undergone quantitative validation, both approaches are described as proposed methodological configurations.

#### 2.6.1. Naive Integration Baseline

As a reference baseline, velocity and position can be estimated by direct numerical integration of acceleration measurements following orientation estimation and gravity compensation [12,13]. In the absence of additional constraints or correction mechanisms, sensor noise and slowly varying bias can accumulate during integration, resulting in increasing trajectory errors over time [4,5]. Naive integration is therefore proposed as a reference condition against which progressively constrained reconstruction strategies can be evaluated in future validation studies [8].

#### 2.6.2. Hybrid Drift-Aware Reconstruction Framework

The proposed hybrid reconstruction framework combines Zero Velocity Updates (ZUPT), an extended Kalman filter (EKF), and the EZERO conceptual adaptive bias estimation scheme [8,12]. Within this configuration, ZUPT provides velocity observations during detected stance phases, the EKF propagates and updates the kinematic state, and EZERO is conceptually intended to inform bias estimation using reliability assessments of candidate low-motion intervals [4]; its update mechanism remains to be specified. Biomechanical constraints related to gait periodicity and stance phases may additionally be incorporated as soft regularization terms to stabilize state estimation without imposing rigid assumptions about normal gait [6,18]. Within the envisioned framework, motion-capture-derived reference trajectories serve as external references for evaluating reconstruction performance and characterizing the discrepancy between estimated and reference motion [7,16]. As described in the residual analysis, this discrepancy represents the total reconstruction residual rather than a direct measure of inertial drift. The framework therefore proposes to examine both residual magnitude and temporal structure, particularly across gait phases. A central hypothesis is that phase-dependent residual patterns may contain information related to movement dynamics in addition to technical and modelling-related error sources. This remains a hypothesis for future investigation rather than an established biomechanical or clinical finding [5].

The envisioned sensor-fusion pipeline consists of the following sequential processing steps (see Figure 1):

1. IMU data acquisition and synchronization;
2. Sensor alignment and gravity compensation;
3. Stance-phase detection;
4. Incorporation of Zero Velocity Updates (ZUPT);
5. EKF-based state propagation and estimation;
6. Conceptual reliability-aware bias estimation using EZERO;
7. Application of biomechanical constraints, where appropriate;
8. Trajectory reconstruction; and
9. Comparison with motion-capture-derived reference trajectories.

This processing chain provides a conceptual structure for investigating how inertial errors may propagate through the reconstruction pipeline and how different correction mechanisms may influence trajectory estimates and their residual characteristics over the gait cycle. The specific implementation, parameterization, and comparative performance of these approaches remain to be established through future empirical evaluation.

##### Biomechanical Constraints

Physiologically inspired constraints are proposed as soft regularization terms to encourage kinematically plausible motion while preserving individual gait variability. These may include weak constraints on step length, velocity, gait periodicity, and overall movement dynamics rather than strict physiological bounds. Their purpose is to reduce physically implausible estimates under noisy measurement conditions without suppressing deviations that may occur in pathological or highly variable gait patterns [9,10]. In the envisioned implementation, the knee may be approximated as a predominantly one-degree-of-freedom hinge joint, whereas the hip may be represented as a three-degree-of-freedom spherical joint. These simplified assumptions provide a computationally tractable representation of major lower-limb degrees of freedom. The constraints could be incorporated into the state-estimation framework as soft penalty terms or probabilistic priors, with their weighting determined through sensitivity analysis rather than fixed a priori. Their formulation and strength would require empirical evaluation to establish whether they improve reconstruction stability without suppressing clinically relevant gait deviations.

#### 2.6.3. Evaluation Strategy

The proposed reconstruction approaches are intended to be evaluated using synthetic and experimental IMU data. Publicly available datasets, including OxIOD, may be considered where the required sensor recordings and suitable reference measurements are available [6]. For datasets containing synchronized IMU and motion-capture (MoCap) recordings, reconstructed trajectories could be compared with MoCap-derived reference trajectories to quantify reconstruction error, assess drift-compensation behavior, and characterize phase-dependent residual structure [7,16]. The evaluation would consider both global error metrics and the temporal distribution of residual discrepancies across gait phases [5]. Candidate performance measures include root mean square error (RMSE), mean absolute error (MAE), trajectory drift magnitude, and deviations in gait parameters such as step length and walking speed. These measures would be computed after appropriate temporal synchronization and coordinate-frame alignment between the reconstructed and reference trajectories. The specific metrics would depend on the available reference signals and the reconstruction outputs. For comparative evaluation, the proposed framework could be assessed against progressively constrained reconstruction strategies, including:

1. Naive double integration;
2. ZUPT-only reconstruction; and
3. EKF-based reconstruction without adaptive bias correction.

These comparison conditions are intended to help isolate the potential contribution of individual correction mechanisms within the proposed hybrid framework. The evaluation remains a proposed component of the conceptual framework; its effectiveness and the contribution of each mechanism have not yet been quantitatively established.

### 2.7. Simulation Protocol

For controlled evaluation, simulation experiments are proposed to investigate the behavior of the framework under systematically varied sensor conditions [13]. Measurement noise, slowly varying bias, and other drift-related characteristics could be varied to represent different operating conditions of wearable IMU systems [4,5]. Multiple simulation configurations would allow sensitivity to noise magnitude, bias dynamics, sampling rate, and initial conditions to be examined rather than relying on a single parameter setting [6]. Synthetic IMU signals could be generated from reference trajectories by incorporating stochastic noise and slowly varying bias components representative of MEMS-based inertial sensing [18,19]. In parallel, publicly available experimental datasets with suitable reference measurements could be considered for subsequent evaluation under realistic locomotor conditions [7,16]. This combined strategy is intended to distinguish controlled investigation of algorithmic behavior from evaluation under naturally occurring motion variability [4,8]. The simulation parameters and assumptions would need to be documented to support reproducibility and interpretation of the results.

#### Proposed Outcome Measures

Potential primary trajectory-level outcome measures include position RMSE, velocity RMSE, endpoint drift, and drift accumulation per gait cycle. Secondary measures may include gait-parameter estimation errors for step length, stride time, cadence, and walking speed. Phase-specific residual characteristics could additionally be quantified for stance, swing, heel-strike, and toe-off intervals using measures such as mean residual magnitude, residual variance, and temporal autocorrelation. These analyses are intended to determine whether residual structure varies systematically across the gait cycle, without assuming a priori that such variation reflects biomechanical or pathological characteristics. Where suitable paired reference measurements are available, agreement between reconstructed trajectories or derived gait parameters and MoCap-based references could be assessed using Bland–Altman analysis and, where the study design supports it, intraclass correlation coefficients (ICCs), with corresponding confidence intervals. Pearson correlation coefficients may additionally be reported to characterize the strength of linear association, but would not be interpreted as a measure of agreement on their own. The choice of statistical method would depend on the outcome, sampling structure, and number of independent participants or trials. Drift accumulation per gait cycle could be defined as the change in displacement error between successive gait-cycle endpoints and normalized by cycle duration. This measure is intended to characterize progressive reconstruction error across repetitive locomotor cycles and to distinguish cumulative error behavior from instantaneous residual magnitude. All outcome measures would require consistent temporal synchronization, coordinate-frame alignment, and clearly defined reference signals. The proposed measures constitute an evaluation plan; their suitability and the framework’s performance remain to be established through empirical analysis.

## 3. Expected Outcomes and Testable Hypotheses

**Figure 3:**
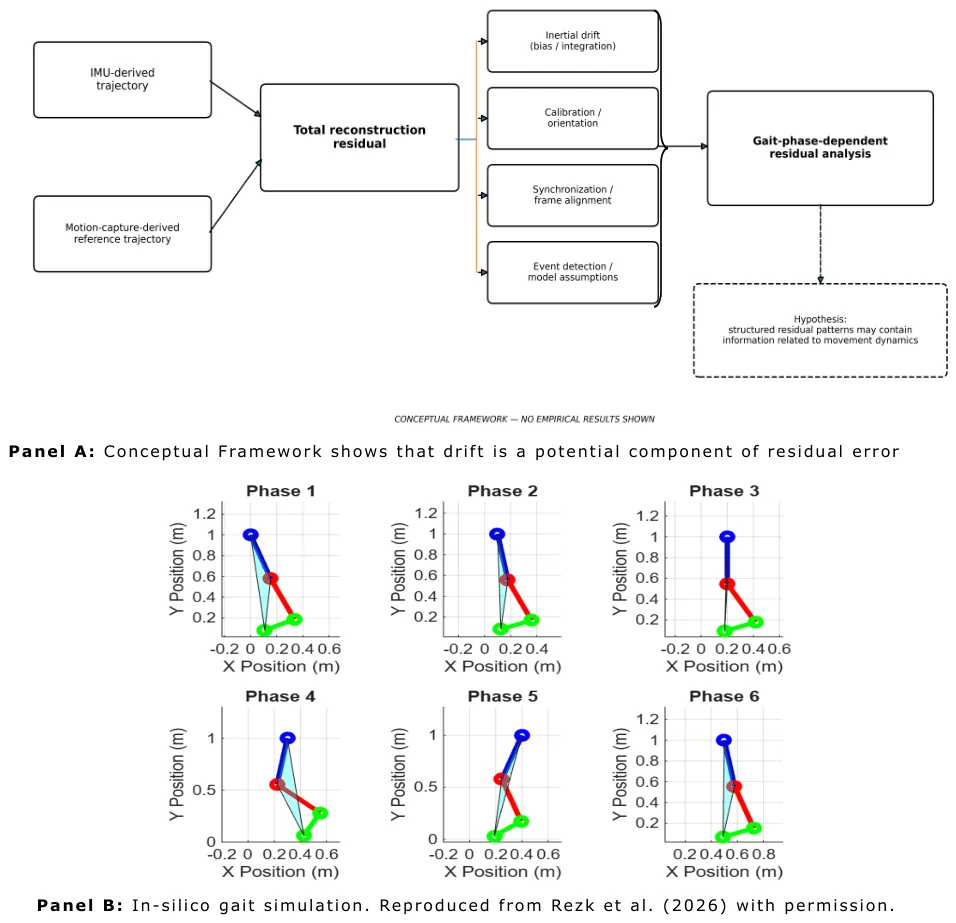
**(Panel A): Conceptual decomposition of total reconstruction residuals in the proposed drift-aware IMU gait framework.** The diagram illustrates potential sources of discrepancy between an IMU-derived trajectory and a motion-capture-derived reference trajectory, including inertial drift, sensor bias, calibration and orientation errors, temporal synchronization and coordinate-frame misalignment, gait-event detection, biomechanical modelling assumptions, and other measurement or processing effects. The total reconstruction residual is therefore not interpreted as a direct measure of inertial drift. The proposed framework aims to characterize the magnitude and temporal structure of this residual across gait phases and to investigate whether reproducible phase-dependent patterns persist after relevant technical sources of error have been characterized or controlled. This decomposition is conceptual and does not represent an empirically validated separation of individual error components. **(Panel B): Conceptual illustration of gait-phase-dependent reconstruction-error behavior across representative stance configurations (1–6).** The six configurations illustrate different foot–ground contact and lower-limb kinematic arrangements during gait rather than the complete set of conventional gait phases. The depicted trajectories are illustrative and do not represent empirical measurements or quantitatively validated simulation results. Ground-contact configurations may provide opportunities for velocity-state correction when valid zero-velocity updates (ZUPTs) are detected, whereas changes in contact configuration and gait-event timing may affect the propagation and correction of inertial estimation errors. The figure illustrates the hypothesis that reconstruction residuals may exhibit phase- or configuration-dependent structure rather than demonstrating an established error pattern. The triangular representation indicates the relative spatial configuration of the foot, knee, and hip checkpoints within the conceptual gait model. Such phase-resolved kinematic information may provide a basis for future investigations of movement stability and gait symmetry, including geometric variability measures such as the Triangular Area Variability (TAV) metric [21].

The following statements represent theoretical expectations and testable hypotheses derived from the proposed framework; they should not be interpreted as empirical findings. They are intended to guide future simulation-based and experimental evaluation using the baseline and reference-based strategies described above. First, unconstrained inertial integration is expected to exhibit increasing reconstruction error as sensor noise and bias accumulate through numerical integration [4,5]. In contrast, the combination of zero-velocity updates (ZUPTs), extended Kalman filter (EKF)-based state estimation, and adaptive bias estimation is hypothesized to reduce cumulative reconstruction error and improve trajectory stability relative to naive integration [8,13]. The magnitude and consistency of any such improvement remain to be established through empirical evaluation. Second, the framework hypothesizes that reconstruction residuals may exhibit systematic temporal variation across gait phases [5]. The in-silico gait simulation shown in Figure 3 conceptually illustrates how reconstruction error could vary across successive gait configurations. It is intended as a visualization of the proposed hypothesis rather than as empirical evidence or a quantitatively validated simulation result. Such phase-dependent residual structure should not be interpreted a priori as evidence of biomechanical or pathological characteristics, as it may also arise from technical factors, including stance detection, sensor alignment, temporal synchronization, filter behavior, and modelling assumptions. Future analyses would therefore need to determine whether these patterns persist after relevant technical sources of error have been characterized or controlled. Third, the framework proposes that residual characteristics may contain information related to movement dynamics beyond global reconstruction error. This is a testable hypothesis rather than an established finding. Future investigations could examine whether residual magnitude, variance, temporal autocorrelation, or other phase-specific characteristics differ systematically across gait conditions or subject groups. Evaluation using controlled synthetic data and experimental datasets with suitable motion-capture-derived reference trajectories could help determine whether the proposed correction mechanisms improve trajectory reconstruction and whether residual structure contains reproducible information related to gait dynamics [7,16]. The potential relevance of these findings to telemedical gait assessment remains a prospective application rather than a demonstrated clinical outcome [9].

### Empirical Illustration of Persistent Gyroscope Components

**Figure 4:**
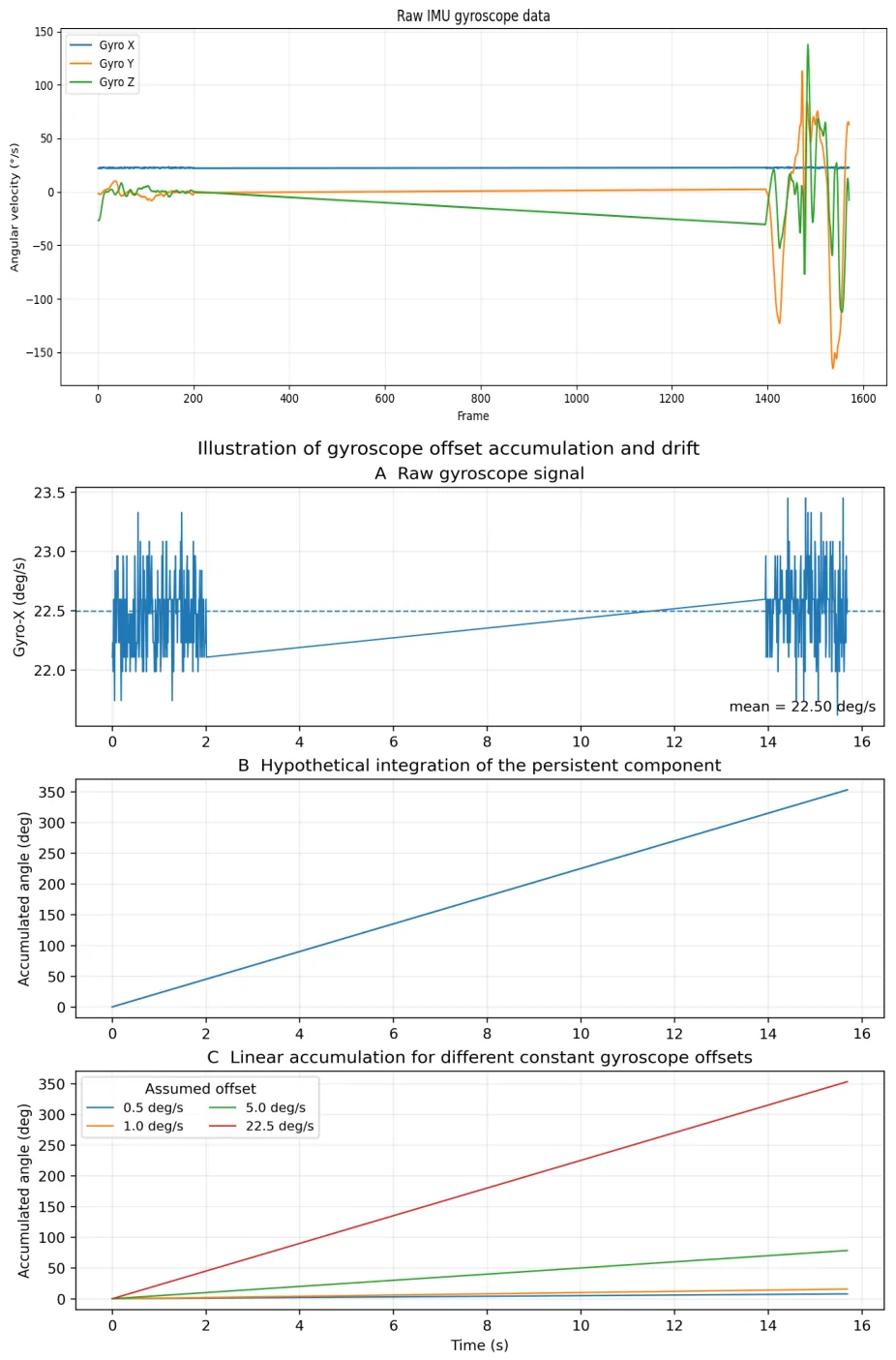
Empirical and theoretical illustration of persistent gyroscope components and their potential relevance to inertial drift in a representative Day 1 gait recording from the dataset reported by Santos et al. [20]. The upper panel shows the raw gyroscope measurements along the *x*-, *y*-, and *z*-axes, with pronounced dynamic angular-rate excursions particularly along the *y*- and *z*-axes and a persistent quasi-DC component of approximately 22–$23^{\circ }/s$ along the *x*-axis. The lower panels illustrate the potential consequences of such a persistent component: (**A**) raw Gyro-X measurements, (**B**) hypothetical angular accumulation obtained by integrating the observed mean component of approximately $22.5^{\circ }/s$, and (**C**) theoretical linear accumulation resulting from different assumed constant gyroscope offsets. Panels (**B**,**C**) represent hypothetical integration scenarios rather than measured orientation drift or experimentally established drift behavior. The observed *x*-axis component is therefore presented as an empirical feature of the raw recording, without assuming that it represents a pure sensor bias or attributing it to a specific underlying mechanism.

To provide a data-driven illustration of the drift-related challenges addressed by the proposed framework, the raw inertial measurement data from a representative Day 1 gait recording from the dataset reported by Santos et al. [20] were additionally inspected. No additional experimental measurement was performed for this analysis. Instead, the original raw gyroscope measurements were revisited to examine their temporal characteristics and to provide an empirical example of persistent angular-rate components that may affect subsequent inertial integration. The raw gyroscope measurements were analyzed directly in their recorded coordinate axes without additional drift correction, bias compensation, or orientation reconstruction. Figure 4 shows the measured angular velocity along the three gyroscope axes over the recording sequence. Pronounced dynamic angular-rate excursions are observed, particularly along the *y*- and *z*-axes, consistent with the rotational dynamics associated with gait. In contrast, the *x*-axis exhibits a persistent quasi-DC component of approximately 22–$23^{\circ }/s$ throughout the recording. A gyroscope measurement can generally be expressed as

(8) $$\omega_{\mathrm{meas}}=\omega_{\mathrm{true}}+b_{g}+n_{g},$$

with $\omega_{\mathrm{meas}}$ representing the measured angular velocity, $\omega_{\mathrm{true}}$ representing the true angular velocity, $b_{g}$ representing the gyroscope bias, and $n_{g}$ representing the measurement noise. A persistent component in the measured signal may originate from a sensor bias, but may also reflect genuine rotational motion, sensor orientation, coordinate-frame effects, calibration characteristics, or other systematic contributions. Consequently, the approximately 22–$23^{\circ }/s$ component observed on the *x*-axis is not interpreted here as a confirmed sensor bias. This distinction is important because the present recording represents a dynamic gait acquisition rather than a dedicated stationary calibration experiment. Without an independently established stationary reference, the available data do not allow the observed *x*-axis component to be uniquely separated into true angular motion and sensor offset. The recording is therefore used as an empirical illustration of a persistent low-frequency component that may become relevant when gyroscope measurements are integrated over time. The potential effect of such a component can nevertheless be illustrated analytically. If, hypothetically, a constant component of $22.5^{\circ }/s$ were entirely attributable to an uncompensated gyroscope offset, direct integration would produce an accumulated angular contribution of

(9) $$\Delta \theta \left(t\right)=b_{g}t,$$

with $b_{g}$ representing the assumed constant angular-rate offset. Under this hypothetical assumption, the accumulated angular error would reach approximately $22.5^{\circ }$ after 1s, $112.5^{\circ }$ after 5s, and $225^{\circ }$ after 10s. These values represent theoretical consequences of constant-offset integration and do not correspond to measured orientation errors in the present recording. The empirical observation and theoretical illustration in Figure 4 motivates the consideration of persistent gyroscope components within the proposed framework. They highlight the potential importance of identifying suitable stationary or quasi-stationary intervals for bias estimation. Accordingly, stationary-event detection, zero-velocity updates (ZUPTs), and a conceptual reliability-aware component such as EZERO are discussed as elements of the proposed drift-aware processing framework. EZERO is intended to assess whether candidate low-motion intervals may provide useful information for inertial bias estimation; however, its operational implementation and the effectiveness of the proposed EZERO/EKF framework are not evaluated in the present study. The analysis presented here is limited to a descriptive inspection of raw gyroscope measurements and theoretical constant-offset integration scenarios. It does not establish sensor bias, quantify actual orientation reconstruction errors, or demonstrate improved estimation performance. Quantitative evaluation against appropriate reference measurements and baseline methods, as well as characterization of sensor bias, repeatability, temperature dependence, and long-term stability, remains future work.

## 4. Discussion and Limitations

The proposed framework presents a conceptual architecture for drift-aware IMU-based gait reconstruction and residual analysis. It builds on established approaches to inertial state estimation, including zero-velocity updates (ZUPTs), Kalman filtering, and bias compensation, while proposing the additional investigation of gait-phase-dependent reconstruction residuals [4,5]. However, the framework remains conceptual, and its performance, robustness, and clinical relevance have not yet been established through quantitative validation. A first methodological limitation concerns the dependence of ZUPT-based correction on reliable stance-phase detection. The assumption that the foot is stationary during stance may not hold consistently in pathological gait, including Parkinsonian shuffling or post-stroke locomotion. Residual foot motion, reduced foot clearance, and irregular stance patterns may therefore compromise the reliability of conventional ZUPT corrections and contribute to state-estimation errors [8]. Future implementations may require adaptive or probabilistic stance-phase inference rather than relying exclusively on fixed threshold-based detection. The suitability of such strategies would need to be evaluated across different gait patterns and sensor placements. A second limitation concerns the assumptions underlying the Extended Kalman Filter (EKF). Its performance depends on the adequacy of the process and measurement models, local linearization, noise characterization, and representation of time-varying sensor bias. These assumptions may become less appropriate during abrupt motion transitions, substantial sensor misalignment, or changes in sensor placement. The effects of these factors on state estimation and reconstruction stability would therefore require systematic sensitivity analysis and empirical evaluation. In particular, the interaction between EKF-based bias estimation and the proposed adaptive bias-correction mechanism (EZERO) needs to be specified and evaluated in a reproducible implementation. A further limitation concerns the interpretation of residual discrepancies between IMU-derived reconstructions and motion-capture-derived reference trajectories. These residuals should not be regarded as direct measures of inertial drift, as they may also reflect calibration and orientation errors, temporal synchronization, coordinate-frame misalignment, gait-event detection, filter behavior, and biomechanical modelling assumptions. Although the proposed framework focuses on the temporal and gait-phase-dependent structure of these residuals, observed patterns may arise from technical or algorithmic factors rather than from underlying biomechanical variability. Future validation studies should therefore characterize or control relevant sources of error before investigating whether residual patterns contain reproducible information related to gait dynamics or pathological movement. The absence of quantitative empirical validation is a central limitation of the current work. The proposed architecture and its expected benefits have not yet been demonstrated using experimental comparisons or systematic simulation results. Publicly available datasets may provide a basis for retrospective evaluation, provided that the required IMU recordings and suitable reference trajectories are available [7,16]. However, the suitability of individual datasets, including OxIOD, must be assessed with respect to sensor configuration, synchronization, and reference-measurement availability before they are used to support trajectory-level validation. Furthermore, laboratory-based datasets may not adequately represent the variability encountered during unsupervised home-based or telemedical gait assessment. The transfer from laboratory reference measurements to IMU-only home monitoring therefore requires separate investigation, particularly because motion-capture reference trajectories would generally not be available during routine deployment. The proposed architecture is conceptually compatible with recursive state estimation and may be suitable for future implementation on mobile or embedded platforms. Nevertheless, real-time performance, computational resource requirements, energy consumption, and robustness during long-term longitudinal operation remain to be evaluated experimentally. Potential applications include longitudinal monitoring of gait characteristics and fall-risk assessment [21,22]. However, phase-resolved residual measures should currently be regarded as exploratory candidate measures rather than validated clinical biomarkers. Their reproducibility, sensitivity to pathological gait, specificity relative to technical confounders, and clinical validity require future empirical investigation. Overall, the proposed framework provides a basis for systematic investigation of drift-aware IMU gait reconstruction and residual analysis. Its potential contribution lies in combining established state-estimation approaches with an explicit examination of reconstruction residuals across gait phases. Demonstrating its practical value will require a reproducible implementation, controlled simulation studies, and experimental validation against suitable reference measurements, followed by evaluation in diverse gait conditions and clinically relevant populations.

### 4.1. Pathological Gait and Assistive Devices

The framework is primarily motivated by gait assessment in healthy adults and individuals with mild to moderate neurological or musculoskeletal impairments. Wearable sensors have been used to objectively quantify gait parameters in patients with Parkinson’s disease, demonstrating their potential for instrumented assessment of disease-related gait abnormalities [23]. In pathological gait patterns, including Parkinsonian shuffling and post-stroke gait, as well as during walking with assistive devices, nominal stance phases may not correspond to truly stationary foot segments. Under these conditions, the conventional zero-velocity assumption may be violated, potentially reducing the reliability of ZUPT-based corrections and introducing systematic estimation errors. Future implementations may therefore require adaptive thresholding or probabilistic stance-detection strategies that account for residual foot motion and altered foot–ground interactions. One possible extension would be to replace fixed ZUPT thresholds with a probabilistic stance classifier that estimates stance likelihood from multiple sensor-derived features, such as estimated foot velocity, acceleration and angular velocity characteristics, and their temporal consistency. Where available, additional information about foot–ground contact could further inform this classification. Such an approach would treat stance detection as a probabilistic estimation problem rather than as a strictly binary decision. However, the reliability of the estimated features and the effects of assistive devices, sensor placement, and atypical movement patterns would need to be considered. The accuracy of wearable IMU-based gait analysis has also been evaluated in healthy female participants, highlighting the importance of population-specific validation [24]. The applicability of these adaptations to pathological gait and assistive device use remains an open empirical question. Accordingly, the proposed framework should be interpreted as a conceptual foundation, with its performance, robustness, and potential clinical relevance in these populations requiring dedicated validation studies.

### 4.2. Control Analyses for Residual Drift Interpretation

The interpretation of phase-dependent reconstruction residuals as potentially movement-related requires dedicated control analyses. Since the total residual may reflect inertial drift as well as calibration, orientation, synchronization, coordinate-frame alignment, gait-event detection, and modelling errors, future investigations should examine whether observed residual structures persist after relevant technical sources of error have been characterized or controlled.

Potential control analyses include the following:

1. **Synthetic trajectories with known bias profiles:** Simulated trajectories with controlled motion characteristics and predefined sensor-noise and bias profiles could be used to examine drift-related effects independently of inter-trial and naturally occurring biomechanical variability.
2. **Gait-phase label perturbation:** Randomized or systematically shifted gait-phase labels could be used as control conditions to assess whether observed residual patterns depend on meaningful temporal organization of the gait cycle.
3. **Stance-detection and event-timing sensitivity:** Sensitivity analyses could examine how variations in stance-detection thresholds and gait-event timing affect residual magnitude, temporal structure, and the resulting reconstruction.
4. **Sensor-placement and orientation perturbation:** Controlled perturbations of sensor placement and orientation could help quantify the contribution of attachment and alignment variability to the observed residuals.
5. **Repeated-trial analyses:** Repeated measurements could be used to assess the reproducibility and reliability of phase-dependent residual characteristics across trials and, where appropriate, across sessions.

Together, these analyses could help distinguish reproducible motion-related residual structure from artifacts associated with sensor configuration, processing parameters, event detection, or reconstruction algorithms. Their implementation would be necessary before residual characteristics could be interpreted as potential indicators of gait dynamics or investigated as candidate clinical measures.

### 4.3. Computational Requirements

The proposed EZERO/EKF pipeline is conceptually compatible with recursive state estimation and may be suitable for implementation on mobile or embedded platforms. The computational requirements are expected to depend on the state dimension, filter formulation, update frequency, and implementation of the associated matrix operations. Although recursive filtering can support online processing, the computational latency, memory requirements, energy consumption, and real-time performance of the proposed framework have not yet been quantitatively evaluated and would require dedicated implementation studies. Cloud-based processing is therefore not assumed to be a strict requirement of the proposed architecture. Nevertheless, cloud infrastructure could support large-scale data storage, longitudinal analysis, and centralized data or model management in future telemedical applications. The relative benefits of local and cloud-based processing would need to be assessed with respect to computational efficiency, data transmission requirements, privacy, and operational reliability.

### 4.4. Potential Telemedicine Applications

The proposed framework may ultimately support telemedical applications such as longitudinal monitoring of gait characteristics in neurological disorders and fall-risk assessment in older adults [21,25,26]. Human gait assessment is also relevant to the evaluation and rehabilitation of neurodegenerative disorders, as gait characteristics can provide information about motor dysfunction and functional impairment [27]. During laboratory-based development and validation, motion-capture-derived reference trajectories could provide an external reference for evaluating reconstruction accuracy, characterizing residual errors, and investigating potentially informative phase-dependent patterns. In home-based applications, however, motion-capture reference trajectories would generally not be available during routine monitoring. Reference-based residual analysis should therefore be regarded primarily as a development and validation strategy for identifying and characterizing reconstruction errors. Following adequate characterization and empirical validation, future studies could investigate whether corresponding features can be estimated reliably from IMU data alone. Variance-based and phase-resolved residual characteristics may provide candidate features that complement conventional spatiotemporal gait parameters, such as cadence, walking speed, and stride length. However, their sensitivity, specificity, reproducibility, and clinical relevance remain undetermined. Prospective empirical studies are therefore required before such features can be considered for clinical interpretation or used as validated biomarkers.

## 5. Conclusions

This perspective presents a conceptual framework for drift-aware IMU-based gait reconstruction and residual analysis. It brings together established inertial estimation strategies, including zero-velocity updates (ZUPTs), extended Kalman filtering, adaptive bias estimation, and biomechanical constraints, within an architecture that incorporates motion-capture-derived reference trajectories [8,12]. Rather than introducing these individual components as novel methods, the framework proposes their integration with a systematic characterization of reconstruction residuals across gait phases. A central proposition is that discrepancies between IMU-derived reconstructions and motion-capture-derived reference trajectories should not be interpreted as direct measures of inertial drift. Instead, total reconstruction residuals may reflect multiple contributing factors, including sensor bias, calibration and orientation errors, temporal synchronization, coordinate-frame alignment, gait-event detection, filtering, and biomechanical modelling assumptions. The proposed phase-dependent residual analysis is intended to investigate whether reproducible residual structures provide information related to movement dynamics beyond global reconstruction error. This proposition remains a testable hypothesis. Phase-dependent residual patterns may arise from technical or algorithmic factors, and their relationship to gait variability, stability, or pathological movement must be established through dedicated control analyses and empirical validation. Accordingly, the proposed framework represents a methodological basis for future investigation rather than a clinically validated diagnostic approach. Datasets containing synchronized IMU recordings and suitable motion-capture-derived reference trajectories could support subsequent evaluation of the reconstruction pipeline and residual-analysis strategy [7,16]. Such studies could assess the reproducibility of residual characteristics, quantify the contribution of individual correction mechanisms, and determine whether residual-based features provide information beyond conventional gait parameters. If validated, selected features could then be investigated for potential use in IMU-only telemedical applications. Overall, the contribution of this work is the formulation of a drift-aware conceptual framework and a set of testable methodological hypotheses. No empirical or clinical validation is claimed in the present perspective. Future studies should implement and quantitatively evaluate the proposed reconstruction pipeline, characterize relevant sources of residual error, and determine whether phase-dependent residual characteristics provide reproducible and clinically meaningful information.

## Data Availability

No new datasets were generated for this perspective. Raw gyroscope measurements from the publicly available dataset described by Santos et al. [20] were descriptively inspected in Section Empirical Illustration of Persistent Gyroscope Components. Publicly available datasets such as OxIOD are discussed only as potential resources for future validation.
